# Molybdenum Oxide Nanoparticle Aggregates Grown by Chemical Vapor Transport

**DOI:** 10.3390/ma15062182

**Published:** 2022-03-16

**Authors:** Yun-Hyuk Choi

**Affiliations:** School of Advanced Materials and Chemical Engineering, Daegu Catholic University, Gyeongsan 38430, Korea; yunhyukchoi@cu.ac.kr

**Keywords:** α-MoO_3_, γ-Mo_4_O_11_, nanoparticles, carbon fiber paper, chemical vapor transport

## Abstract

In this study, the advanced chemical vapor transport (CVT) method in combination with the quenching effect is introduced for creating molybdenum oxide nanoparticle arrays, composed of the hierarchical structure of fine nanoparticles (NPs), which are vertically grown with a homogeneous coverage on the individual carbon fibers of carbon fiber paper (CFP) substrates. The obtained molybdenum oxide NPs hold a metastable high-temperature γ-Mo_4_O_11_ phase along with a stable α-MoO_3_ phase by the quenching effect. Furthermore, such a quenching effect forms thinner and smaller nanoparticle aggregates by suppressing the growth and coalescence of primary particles. The molybdenum oxide nanoparticle aggregates are prepared using two different types of precursors: MoO_3_ and a 1:1 (mol/mol) mixture of MoO_3_ and activated carbon. The results characterized using X-ray diffraction, Raman spectroscopy, X-ray photoelectron spectroscopy, and Fourier-transform infrared spectroscopy show that the relative amount of α-MoO_3_ to γ-Mo_4_O_11_ within the prepared NPs is dependent on the precursor type; a lower amount of α-MoO_3_ to γ-Mo_4_O_11_ is obtained in the NPs prepared using the mixed precursor of MoO_3_ and carbon. This processing–structure landscape study can serve as the groundwork for the development of high-performance nanomaterials in various electronic and catalytic applications.

## 1. Introduction

Nanoscale materials have attracted intensive attention owing to their high surface area and chemical activities different from their bulk counterparts, relevant to electronics, catalysis, sensors, and energy storage applications [1,2,3,4,5,6,7,8]. Among various synthesis methods for those materials, the chemical vapor deposition (CVD) technique chemical vapor transport has been extensively used for the large-scale synthesis of uniform-sized nanomaterials [4,9,10,11]. However, in most cases, the obtained nanomaterials often suffered from poor size and shape homogeneity, with sparse substrate coverage, which are attributed to several concurrent reactions [10,11]. Particularly in the case of growth of two-dimensional (2D) nanosheets, the dominant growth of basal planes with low chemical and catalytic activities was revealed as major drawbacks of the conventional CVD technique for applications. In previous work, the novel stepwise CVD process which enabled the large-scale synthesis of the homogeneously sized and well-distributed nanoparticles (NPs) was proposed as a solution to the problems mentioned above [9]. However, the slow rate of natural furnace cooling process from the high processing temperature of 850 °C in the synthesis protocol produced somewhat thick and large-sized NPs.

Therefore, in this work, we modify the cooling time to be extremely short in the CVD process (hereafter referred to as chemical vapor transport) in order to obtain thinner and smaller nanoparticle aggregates (secondary particles) by suppressing the growth and coalescence of primary particles. Such a quenching effect from high temperature to room temperature for the nanoparticle deposition is achieved by placing the position of the substrate outside the tube furnace. Interestingly, the obtained nanoparticle aggregates hold a metastable high-temperature phase together with a stable phase within their microstructure. Their morphology exhibits the hierarchical structure composed of fine primary NPs. In addition, we deposited the molybdenum oxide nanoparticle aggregates onto conductive carbon fiber papers (CFPs) with high surface area by considering various electronic and catalytic applications such as gas sensors, supercapacitors, photocatalysts, environmental catalysts, and electrocatalysts.

Molybdenum oxide, chosen as a case material in this work, is a transition metal oxide with a versatile oxidation state and crystal structure, which has been widely used for various applications in electronics, catalysis, sensors, and electrochromic and energy storage systems [12,13,14,15,16]. In particular, molybdenum oxide possesses a wide range of non-stoichiometries from full stoichiometric MoO_3_ (Mo^6+^ ions) to reduced MoO_3−x_ (2 < x < 3) (mainly Mo^5+^ ions and mixed valence) and finally to semi-metallic MoO_2_ (Mo^4+^ ions) with varying oxygen vacancies [12]. The stoichiometric MoO_3_ reveals diverse crystal structures: orthorhombic α-MoO_3_ (space group P_nma_) as the thermodynamically most stable phase and monoclinic β-MoO_3_ (P2_1_/c), ε-MoO_3_ (high-pressure phase, MoO_3_-II, P2_1_/m), and hexagonal h-MoO_3_ (P6_3_/m) as the metastable polymorphs [12,14]. The non-stoichiometric MoO_x_ displays more diverse sub-oxide structures with average valences between Mo^6+^ and Mo^4+^ (Mo_18_O_52_, Mo_17_O_47_, Mo_9_O_26_, Mo_8_O_23_, Mo_5_O_14_, and Mo_4_O_11_) [12]. Among them, the metastable Magnéli phase Mo_4_O_11_ is one of the most commonly observed structures and is found either as low-temperature monoclinic η-phase or high-temperature orthorhombic γ-phase (or called α-phase) [12,15,17,18]. It is known that the reduction process of MoO_3_ involves the formation of Mo_4_O_11_ as the intermediate product [12,17]. In this regard, herein, we investigate structure and phase of molybdenum oxide NPs in terms of the processing–structure relationship.

Recently, various methods such as hydrothermal, sol–gel, spray pyrolysis, thermal evaporation, chemical bath deposition, and templated processes have been applied to the preparation of molybdenum oxides (mainly MoO_3_) [19,20,21,22,23,24]. Furthermore, the conventional CVD processes of MoO_3_ have been reported [25,26,27,28]. Through such CVD processes, it was easy to obtain complete amorphous or single-phased polycrystalline/single-crystalline molybdenum oxides. Otherwise, molybdenum oxides with randomly mixed stable and metastable phases were obtained by such processes, in which it was very difficult to control the phases. In contrast to thermodynamic processing, the CVD method developed in this work involves a more kinetic component. The novelty of this study can be found in the provision of an idea that can intentionally control the material phase through the facile operation of the processing.

## 2. Experimental

### 2.1. Deposition of Molybdenum Oxide Nanoparticle Aggregates

The chemical vapor transport (CVT) processes were performed using a horizontal cold-wall quartz tube (32 mm in diameter and 600 mm in length) furnace, equipped with gas flow controls. The furnace length was 400 mm, and both ends of the quartz tube were projected to the furnace outside each 100 mm. To deposit molybdenum oxide NPs, two different powder precursors, MoO_3_ powder (Sigma-Aldrich, St. Louis, MI, USA, ≥99.5% purity) and a 1:1 (mol/mol) mixture of MoO_3_ powder and activated carbon, were attempted. Each powder precursor was placed with the same amount of 15.0 mg within an alumina boat, which was located at the center of the tube. A bare carbon fiber paper (CFP, Toray paper 120) substrate with dimensions of 2 cm × 1 cm adhered using a 3M double-sided thermal tape to the top of the wall inside the quartz tube was placed downstream from the precursor source and located 50 mm outside of the furnace in order to induce the quenching effect. After an initial Ar purge for 20 min, the precursor powder was heated to 850 °C at a ramp rate of 20 °C/min and transported under a 30 cc/min Ar flow at 1 atm. After the temperature was held at 850 °C for 10 min, the furnace cooled naturally to room temperature.

### 2.2. Structural Characterization

The morphology of the samples was examined by field-emission scanning electron microscopy (FE-SEM, Tokyo, Japan) using a Hitachi S-4800 instrument. The elemental composition of the samples was examined by energy-dispersive X-ray spectroscopy (EDS) coupled to the FE-SEM system. Phase assignment was carried out with the help of X-ray diffraction (XRD) using a Rigaku D/Max-2500 equipped with a Cu Kα source (λ = 1.5406 Å), as well as confocal Raman microprobe analysis using a Horiba XploRA instrument (Kyoto, Japan). Raman spectra were collected with excitation from the 532 nm line of an air-cooled solid laser. The chemical composition and oxidation state of the samples were investigated by X-ray photoelectron spectroscopy (XPS, K-Alpha, Thermo Scientific, Waltham, MA, USA) with micro-focused monochromatized Al Kα radiation (1486.6 eV). The energy calibration was achieved by setting the hydrocarbon C 1*s* line at 284.80 eV. The surface states of the samples were analyzed by Fourier-transform infrared spectroscopy (FTIR, Bruker ALPHA 2, Billerica, MA, USA). The morphology and crystal structure of the samples were further observed by high-resolution transmission electron microscopy (TEM, Titan G2 ChemiSTEM Cs Probe, FEI Company, Hillsboro, OR, USA) operated at an accelerating voltage of 200 kV.

## 3. Results and Discussion

**Figure 1:**
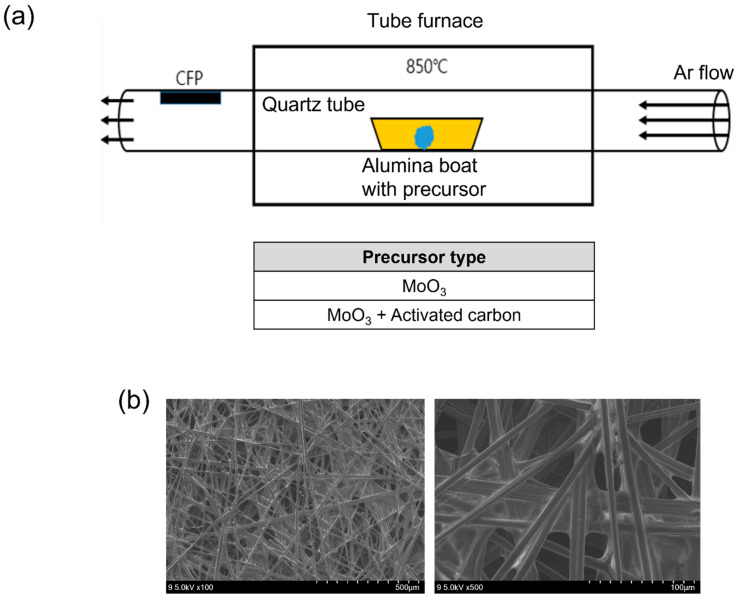
(**a**) Illustration of the chemical vapor transport (CVT) process combined with the quenching effect achieved by placing the position of the substrate outside the tube furnace, used to grow molybdenum oxide nanoparticle aggregates on carbon fiber paper (CFP) substrates using two different types of precursors. (**b**) FE-SEM images of bare CFP.

**Figure 2:**
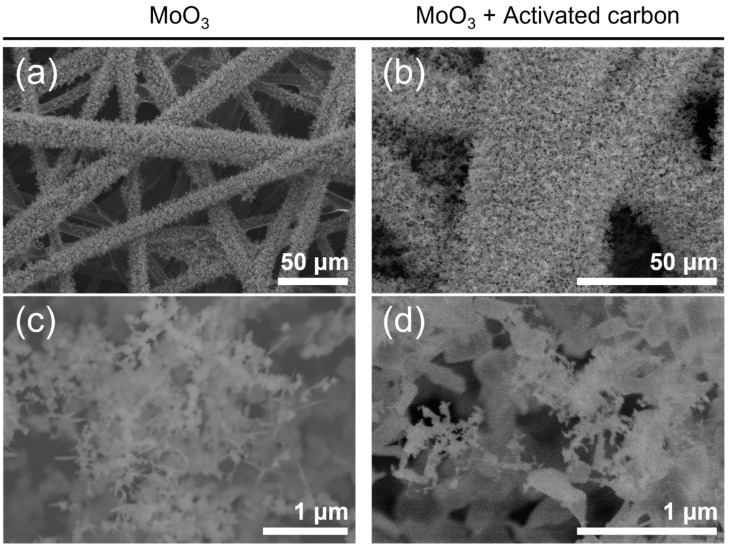
FE-SEM images of the molybdenum oxide nanoparticle aggregates prepared on CFP substrates by the CVT method using two different types of precursors: (**a**,**c**) MoO_3_ and (**b**,**d**) 1:1 (mol/mol) mixture of MoO_3_ and activated carbon. (**c**,**d**) High-magnification FE-SEM images.

The CVT processes were performed to prepare the molybdenum oxide nanoparticle aggregates on CFP, as illustrated in Figure 1a. The molybdenum oxide nanoparticle aggregates were deposited onto CFP adhered to the top of the quartz tube inside by the vapor transport of precursor heated to 850 °C. The deposition was attempted using two different powder precursors, MoO_3_ and a 1:1 (mol/mol) mixture of MoO_3_ and activated carbon, referred to as ‘M’ and ‘MC’, respectively. The use of the mixed precursor of MoO_3_ and activated carbon was expected to induce the reduction effect (defect or non-stoichiometry) by carbon species for the deposition of molybdenum oxide. Since the melting and boiling points of α-MoO_3_ are known to be 795 and 1155 °C, respectively, the processing temperature for the vapor transport of precursors was set to 850 °C. The FE-SEM images in Figure 2 show the homogeneous coverage and vertical growth orientation of molybdenum oxide NPs on the individual carbon fibers of CFP, similarly with respect to the products obtained using two different precursors. The morphology of bare CFP, shown in Figure 1b, is contrasted. All the prepared nanoparticle aggregates revealed a fluffy and hierarchical structure composed of fine primary NPs, which were vertically aligned from the supporting carbon fibers of CFP. The primary NPs composing aggregates came in different sizes depending on the type of precursor: 100 nm for ‘M’ and 250 nm for ‘MC’ in diameter. Interestingly, when ‘MC’ was used as a precursor, only carbon powder was found to remain in an alumina boat with no transport after processing.

**Figure 3:**
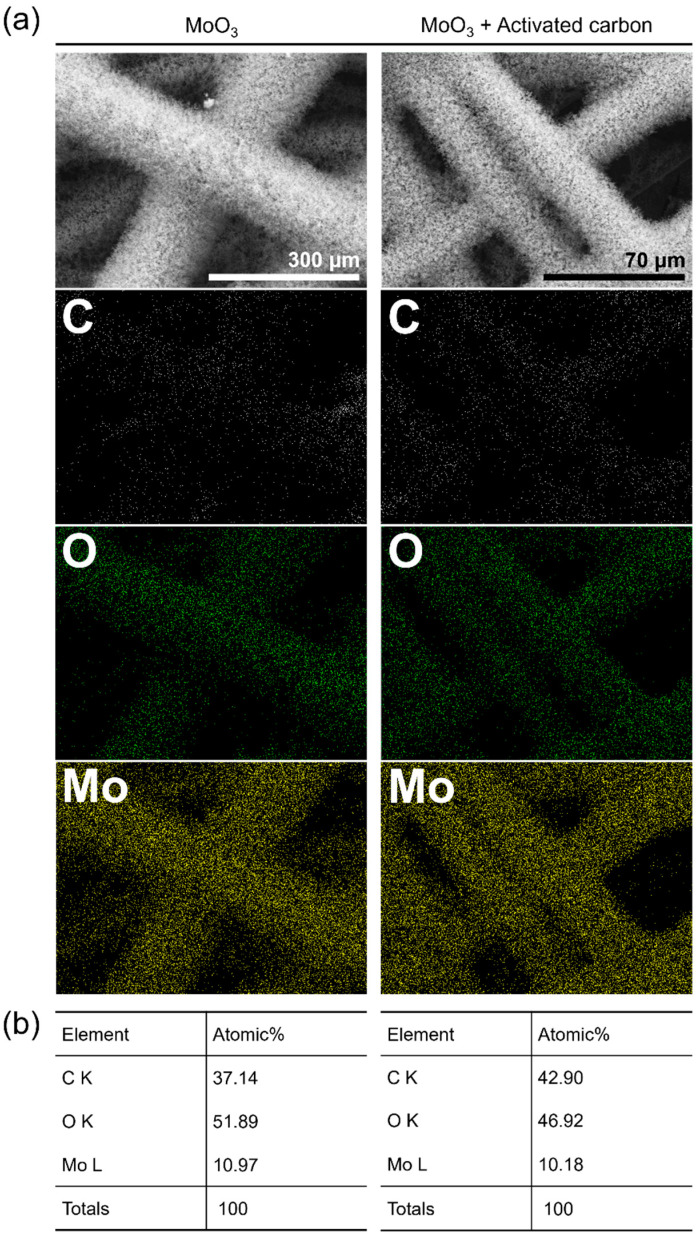
(**a**) EDS elemental maps and (**b**) atomic ratios of C, O, and Mo acquired for the molybdenum oxide nanoparticle aggregates prepared on CFP substrates by the CVT method using two different types of precursors: MoO_3_ and 1:1 (mol/mol) mixture of MoO_3_ and activated carbon.

Figure 3a exhibits EDS elemental maps of C, O, and Mo acquired for the samples. It once again verifies the homogeneous coverage of molybdenum oxide NPs on the individual carbon fibers of CFP for both samples. The measured atomic ratios are presented in Figure 3b. The molybdenum oxide nanoparticle aggregates prepared on CFP using ‘M’ exhibited a slightly higher O/Mo ratio compared with those prepared using ‘MC’.

**Figure 4:**
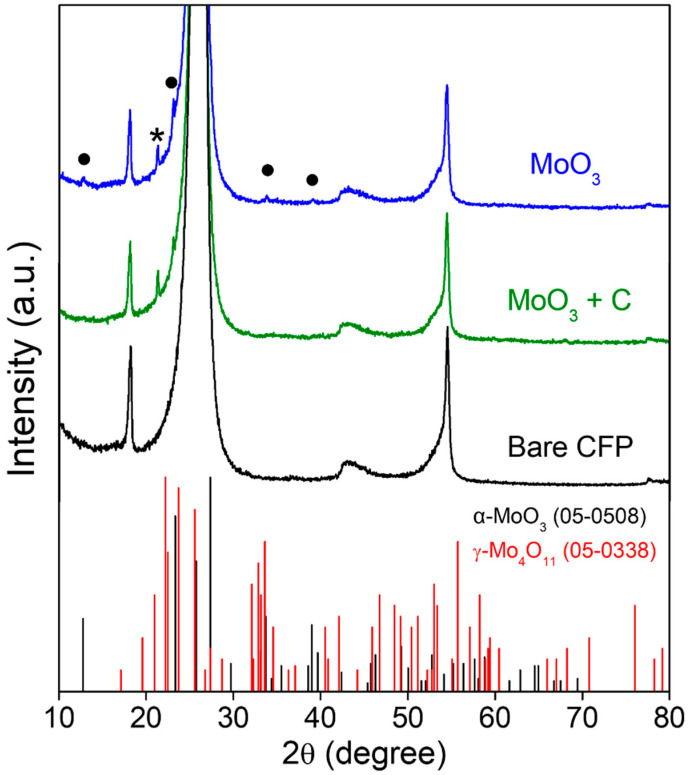
XRD patterns of the molybdenum oxide nanoparticle aggregates prepared on CFP substrates by the CVT method using two different types of precursors: MoO_3_ and 1:1 (mol/mol) mixture of MoO_3_ and activated carbon and that of bare CFP (“•” and “*”represent the peaks arising from α-MoO_3_ and γ-Mo_4_O_11_ phases, respectively). The bottom bars are JCPDS standards for orthorhombic α-MoO_3_ (no. 05-0508) and γ-Mo_4_O_11_ (no. 05-0338).

The XRD analysis was performed for all samples, and the acquired diffraction patterns are shown in Figure 4. The peaks corresponding to α-MoO_3_ and γ-Mo_4_O_11_ are revealed for the molybdenum oxide nanoparticle aggregates formed on CFP using ‘M’ according to the Joint Committee on Powder Diffraction Standards (JCPDS) no. 05-0508 and 05-0338, respectively. For the sample prepared using ‘MC’, the peaks arising from α-MoO_3_ weakened, while the main peak arising from γ-Mo_4_O_11_ was slightly strengthened compared with the sample prepared using ‘M’. However, the peaks arising from molybdenum oxide were hard to observe because of the strong peaks arising from CFP substrate. Hence, we used Raman spectroscopy to investigate the crystal phase of molybdenum oxide nanoparticle aggregates more clearly.

**Figure 5:**
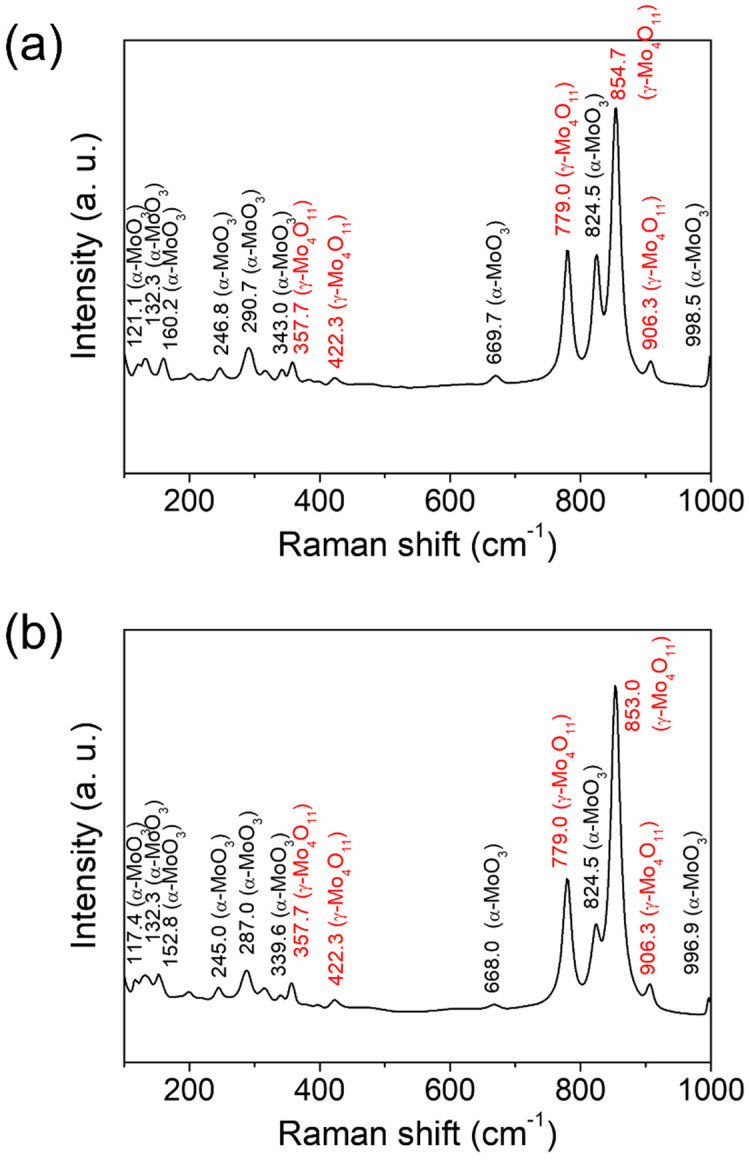
Raman spectra of the molybdenum oxide nanoparticle aggregates prepared on CFP substrates by the CVT method using two different types of precursors: (**a**) MoO_3_ and (**b**) 1:1 (mol/mol) mixture of MoO_3_ and activated carbon.

Figure 5 shows the Raman spectra of the molybdenum oxide nanoparticle aggregates prepared using two different types of precursors. Noticeably, the spectra arising from orthorhombic α-MoO_3_ and γ-Mo_4_O_11_ were acquired for both samples, which correspond to the reported Raman bands [9,29,30,31]. The main bands of Raman spectra arising from the two phases of molybdenum oxide were observed at 669.7 cm^−1^ (B_2g_, B_3g_), 824.5 cm^−1^ (A_g_, B_1g_), and 998.5 cm^−1^ (A_g_, B_1g_) for α-MoO_3_ and at 779.0, 854.7, and 906.3 cm^−1^ for γ-Mo_4_O_11_. The relative amounts of α-MoO_3_ and γ-Mo_4_O_11_ within the NPs can be identified by comparing the relative peak intensities (I(α-MoO_3_)/I(γ-Mo_4_O_11_)) for both samples. The I(α-MoO_3_)/I(γ-Mo_4_O_11_) ratio was obtained by Gaussian fitting of the intensity (integrated area) ratio between the two primary peaks of 824.5 cm^−1^ (A_g_, B_1g_) for α-MoO_3_ and 854.7 cm^−1^ for γ-Mo_4_O_11_. As a result, it can be found that the amount of γ-Mo_4_O_11_ was relatively more than that of α-MoO_3_ with an I(α-MoO_3_)/I(γ-Mo_4_O_11_) ratio of 0.45 for the molybdenum oxide NPs prepared using ‘M’ (Figure 5a). For the sample prepared using ‘MC’, the amount of α-MoO_3_ decreased further compared to that of γ-Mo_4_O_11_, with an I(α-MoO_3_)/I(γ-Mo_4_O_11_) ratio of 0.28 (Figure 5b). These results correspond to the aforementioned changing trend in atomic ratio measured by EDS elemental analysis (the O/Mo ratio of α-MoO_3_ is slightly higher than that of γ-Mo_4_O_11_) and phase variation identified by XRD analysis between both samples. The lowest amount of α-MoO_3_ phase, with the higher O/Mo ratio compared with that of γ-Mo_4_O_11_, for the sample prepared using ‘MC’ is believed to have been due to the reduction effect by the activated carbon of precursor. While the orthorhombic α-MoO_3_ is the major stable phase of molybdenum oxide, the crystal structure of the metastable Magnéli phase Mo_4_O_11_ can be found either as a low-temperature monoclinic η-phase or high-temperature orthorhombic γ-phase (also called α-phase) [12,15,17,18]. In our case, the quenching effect from the high temperature of 850 °C to room temperature for deposition onto the substrate located outside of the tube furnace was considered to lead to the formation of high-temperature γ-Mo_4_O_11_ phase along with the stable α-MoO_3_ phase within the NPs.

**Figure 6:**
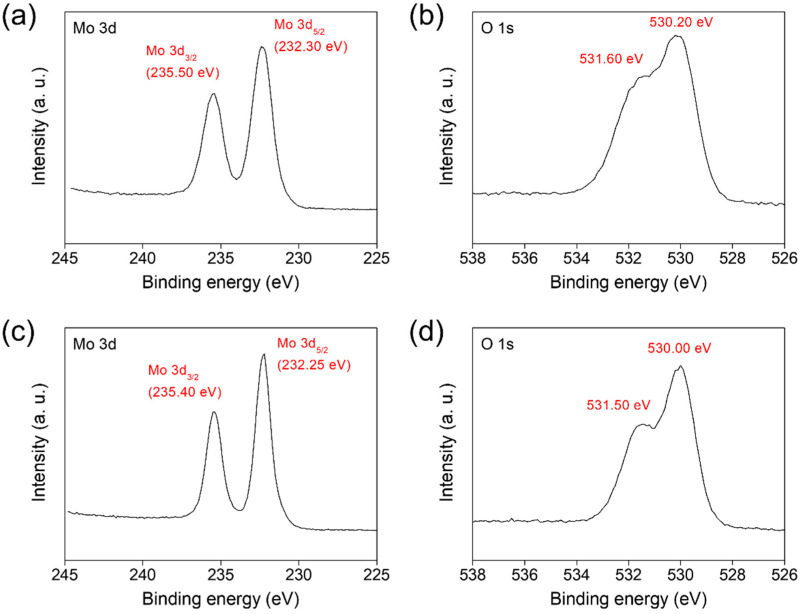
XPS spectra of Mo 3*d* and O 1*s* acquired for the molybdenum oxide nanoparticle aggregates prepared on CFP substrates by the CVT method using two different types of precursors: (**a**,**b**) MoO_3_ and (**c**,**d**) 1:1 (mol/mol) mixture of MoO_3_ and activated carbon.

The surface chemical states of the molybdenum oxide nanoparticle aggregates prepared using two different types of precursors were investigated by XPS. As shown in Figure 6a,c, similar Mo 3*d* spectra for both samples were acquired, revealing the Mo 3*d*_3/2_ peak at 235.40–235.50 eV, Mo 3*d*_5/2_ peak at 232.25–232.30 eV, and Mo 3*d*_3/2_–Mo 3*d*_5/2_ separation of 3.15–3.20 eV. Those spectral characteristics indicate that the molybdenum oxide NPs consisted mainly of Mo^6+^ [15,18,32,33]. In other words, they reflected Mo^6+^ of the γ-Mo_4_O_11_ phase and α-MoO_3_ phase, although the γ-Mo_4_O_11_ phase contained a small dose of the Mo^5+^ component, along with a large amount of Mo^6+^ component [15,18]. The O 1*s* spectra provide more interesting findings. The peaks were observed at 531.60 and 530.20 eV for the O 1*s* spectra of both samples, originating from the lattice oxygens of α-MoO_3_ and γ-Mo_4_O_11_, respectively (Figure 6b,d) [9,24]. Noticeably, the relative intensity of the peak at 531.60 eV to the peak at 530.20 eV decreased according to the type of precursor: ‘M’ (Figure 6b) → ‘MC’ (Figure 6d). This indicates that the relative amount of α-MoO_3_ to γ-Mo_4_O_11_ within the NPs decreased according to the change in precursor type. This corresponds well to the preceding results characterized using various tools including Raman spectroscopy.

**Figure 7:**
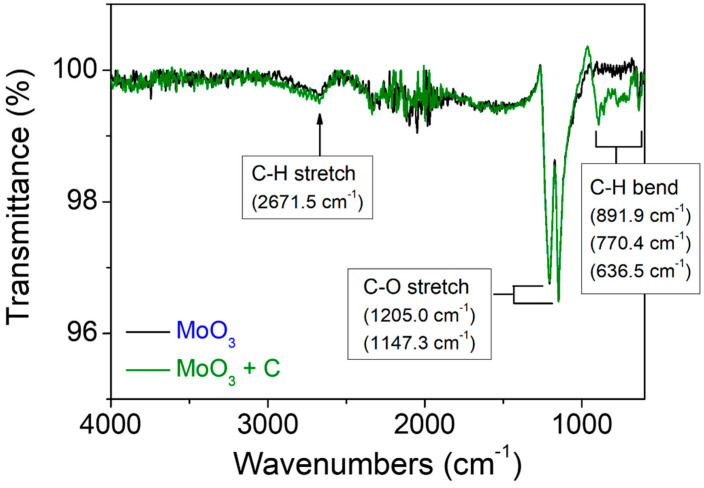
FTIR transmittance spectra of the molybdenum oxide nanoparticle aggregates prepared on CFP substrates by the CVT method using two different types of precursors: MoO_3_ and 1:1 (mol/mol) mixture of MoO_3_ and activated carbon.

The surface states of the molybdenum oxide nanoparticle aggregates prepared using two different types of precursors were further examined by FTIR. Figure 7 shows the FTIR transmittance spectra acquired for the two samples. The peaks corresponding to C–H stretching (2671.5 cm^−1^), C–O stretching (1205.0 cm^−1^ and 1147.3 cm^−1^), and C–H bending (636.5 cm^−1^) were obtained for both samples. In particular, it is worth noting that the small peaks corresponding to C–H bending at 891.9 cm^−1^ and 770.4 cm^−1^ were observed only for the sample prepared using ‘MC’. This indicates that the molybdenum oxide nanoparticle aggregates prepared using ‘MC’ contained more carbon species on their surface compared with those prepared using ‘M’.

**Figure 8:**
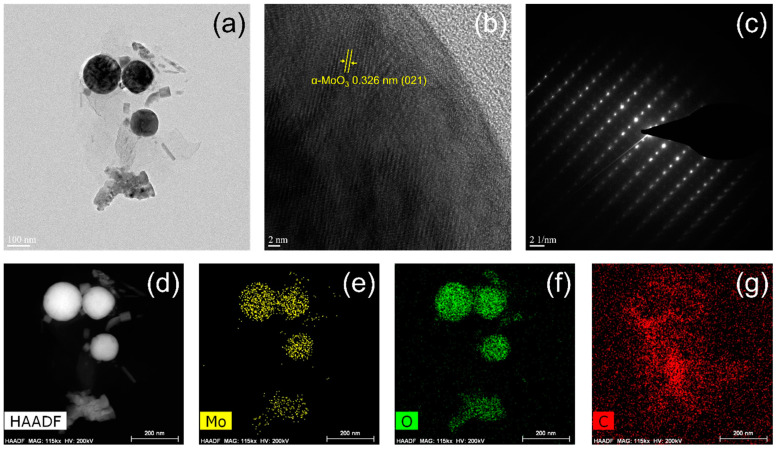
(**a**) Low-magnification and (**b**) high-resolution TEM images, (**c**) SAED pattern, (**d**) HAADF STEM image, and EDS mapping images of (**e**) Mo, (**f**) O, and (**g**) C, acquired for the molybdenum oxide nanoparticles (NPs) prepared by the CVT method using MoO_3_ precursor. The NPs were harvested from the CFP substrates for the characterization.

**Figure 9:**
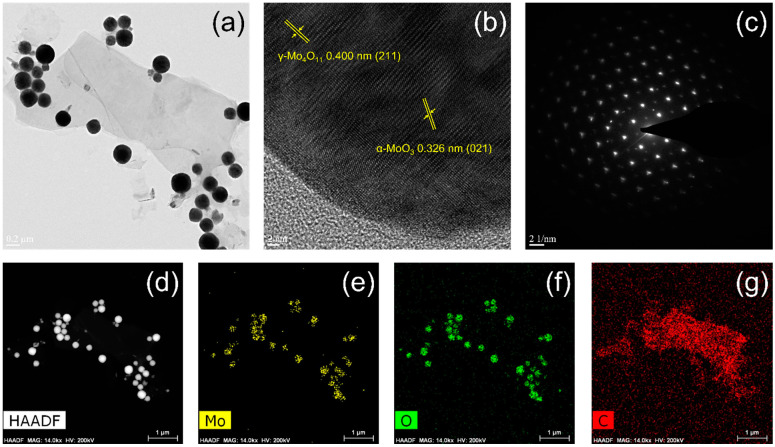
(**a**) Low-magnification and (**b**) high-resolution TEM images, (**c**) SAED pattern, (**d**) HAADF STEM image, and EDS mapping images of (**e**) Mo, (**f**) O, and (**g**) C, acquired for the molybdenum oxide NPs prepared by the CVT method using 1:1 (mol/mol) mixed precursor of MoO_3_ and activated carbon. The NPs were harvested from the CFP substrates for the characterization.

Figure 8 and Figure 9 show low-magnification and high-resolution TEM images, selected-area electron diffraction (SAED) patterns, high-angle annular dark-field (HAADF) scanning transmission electron microscopy (STEM) images, and energy-dispersive spectroscopy (EDS) mapping images of Mo, O, and C, acquired for both samples,. The NPs were harvested from the CFP substrates by ultrasonication in toluene for characterization. In Figure 8a and Figure 9a, the spherical NPs observed for both samples exhibit size differences depending on the type of precursor, which coincides with the FE-SEM results in Figure 2 (100 nm for ‘M’ and 250 nm for ‘MC’ in diameter). The TEM lattice fringe image of the molybdenum oxide nanoparticle prepared by the CVT method using ‘M’ in Figure 8b reveals dominant (021) planes of the α-MoO_3_ phase, whereas those of the γ-Mo_4_O_11_ phase are hard to observe. The corresponding SAED pattern shows a highly ordered orthorhombic crystal lattice structure of the nanoparticle (Figure 8c). Meanwhile, the TEM lattice fringe image of the molybdenum oxide nanoparticle prepared by the CVT method using ‘MC’ in Figure 9b represents (211) planes of the γ-Mo_4_O_11_ phase, as well as (021) planes of the α-MoO_3_ phase, probably owing to relatively higher content of the γ-Mo_4_O_11_ phase within the nanoparticle as verified earlier. Its SAED pattern also exhibits a highly ordered orthorhombic structure in Figure 9c. The HAADF STEM and EDS mapping images of both NPs reveal the homogeneous distribution of Mo and O elements throughout the NPs (Figure 8d–g and Figure 9d–g).

This work addressed the advanced preparation method of molybdenum oxide nanoparticle aggregates, modulating their structure and phase. These results can serve as the groundwork for the development of high-performance nanomaterials in various electronic and catalytic applications such as gas sensors, supercapacitors, photocatalysts, environmental catalysts, and electrocatalysts [19,20,21,22,23,24,25,26,27].

## 4. Conclusions

The thin and small-sized molybdenum oxide nanoparticle aggregates were uniformly deposited on CFP substrates by the CVT method, combined with a quenching effect achieved by placing the position of the substrate outside the tube furnace. The obtained molyndenum oxide nanoparticle arrays revealed the hierarchical structure of fine NPs, which were vertically grown with a homogeneous coverage on the individual carbon fibers of CFP substrates. Furthermore, their phase held a metastable high-temperature γ-Mo_4_O_11_ phase along with a stable α-MoO_3_ phase. The influence of precursor type on the formation of molybdenum oxide nanoparticle aggregates by the advanced CVT technique was investigated using two different types of precursors: MoO_3_ (‘M’) and a 1:1(mol/mol) mixture of MoO_3_ and activated carbon (‘MC’). The results characterized using XRD, Raman spectroscopy, XPS, and FTIR showed that the relative amount of α-MoO_3_ to γ-Mo_4_O_11_ within the NPs decreased according to the type of precursor as follows: ‘M’ → ‘MC’.

## Data Availability

Not applicable.
